# Effect of concentration and thickness on tribological performance and stress distribution of polyvinyl alcohol coatings on aluminum substrates

**DOI:** 10.1039/d5ra06231d

**Published:** 2025-10-29

**Authors:** Sung-Jun Lee, Hye-Min Kwon, Chang-Lae Kim

**Affiliations:** a Department of Mechanical Engineering, Chosun University Gwangju 61452 Republic of Korea kimcl@chosun.ac.kr

## Abstract

This study investigated the tribological performance and stress distribution mechanisms of pure polyvinyl alcohol (PVA) coatings on aluminum (Al) 5052 alloy substrates. PVA coatings were prepared through solution casting at concentrations ranging from 0.1 to 10 wt%, resulting in coating thicknesses from 0.32 to 307 μm. Tribological tests revealed that the coatings prepared at 5 wt% with thicknesses of 44.2–73.8 μm exhibited optimal performance, achieving friction coefficients as low as 0.157–0.158, representing a 76% reduction compared to bare aluminum (0.67). Long-term durability tests under varying loads (50–100 mN) for 10 000 cycles demonstrated that the optimized coatings maintained superior performance across all conditions. Wear track analysis showed that thin coatings below 7 μm suffered from substrate exposure through abrasive wear, whereas excessively thick coatings above 150 μm experienced delamination and severe plastic deformation. Finite element analysis revealed that the PVA coatings reduced the von Mises stress on both the counter tip and substrate by 31–51% through effective stress redistribution. The low elastic modulus of PVA (1.4 GPa) compared to that of aluminum (70 GPa) enables load distribution over wider contact areas, preventing localized damage. The surface roughness decreased from 0.103 μm for bare Al to 0.033 μm for optimally coated specimens, contributing to improved tribological behavior. These findings demonstrate that pure PVA coatings can serve as effective protective layers for aluminum components without the complexity of composite systems, with critical parameters identified for their practical applications.

## Abstract 2

This study investigated the tribological performance and stress distribution mechanisms of pure polyvinyl alcohol (PVA) coatings on aluminum (Al) 5052 alloy substrates.

## Graphical abstract

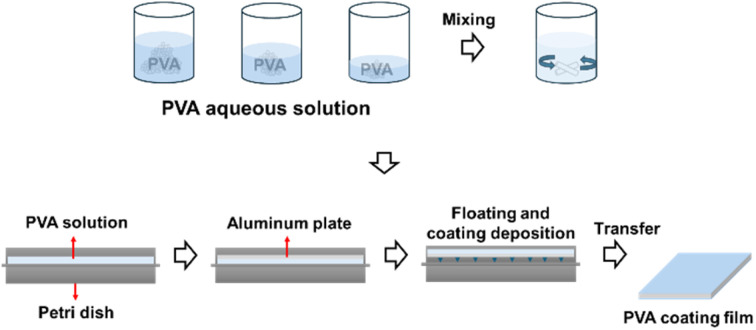

## 1. Introduction

Energy losses due to friction and wear in mechanical systems account for approximately 23% of global energy consumption, resulting in economic losses exceeding $500 billion annually in industrialized nations.^1^ Aluminum alloys are widely used in the aerospace, automotive, and marine industries owing to their light weight, excellent thermal conductivity, and high specific strength. However, their relatively low hardness and wear resistance limit their tribological applications.^2,3^ Among the various surface treatment technologies for improving the tribological performance of aluminum surfaces, polymer coatings have attracted attention owing to their low friction coefficients, self-lubricating properties, excellent conformability, and relatively simple processing.^4^

Polyvinyl alcohol (PVA) has several advantages as a tribological coating material. PVA contains high-density hydroxyl groups on its main chain, enabling the formation of extensive hydrogen bonding networks, which simultaneously improve its mechanical properties and surface adhesion.^5^ The semi-crystalline structure of PVA allows property control through processing conditions, and its water-soluble nature enables environmentally friendly coating. According to studies by Kosukegawa *et al.*, PVA hydrogels exhibit low friction coefficients below 0.1 when in contact with SUS 316L stainless steel, which is attributed to the adsorption–exclusion mechanism of PVA chains.^6^

Recent research on improving the tribological performance of PVA has primarily focused on the development of composites. Min *et al.* developed hierarchically structured composite coatings of graphene oxide (GO) and PVA inspired by natural nacre structures, reporting that GO/PVA composite coatings achieved a low friction coefficient of 0.085 and significantly reduced wear rates compared to pure GO coatings.^7^ Similarly, GO/PVA/borax composite coatings mimicking “bricks-and-mortar’’ structures achieved an ultralow friction coefficient of 0.021 in aqueous environments, showing a 97.86% friction reduction compared to the substrate.^8^

However, these composite systems have some limitations. The synthesis and dispersion processes of GO are complex, which restricts large-scale production. The uniform dispersion of GO requires additional processes, such as ultrasonic treatment, surface modification, and pH adjustment. These factors lead to increased production costs and difficulties in quality control.^9^ Furthermore, there is no consensus on the optimal GO loading, with various studies reporting a wide range of concentrations for GO. This suggests that the interfacial properties between the GO and PVA matrix are highly sensitive to the manufacturing conditions. Most studies were conducted under single test conditions, lacking comprehensive performance evaluation for practical applications, which represents another significant limitation.

Research on pure PVA coating systems is limited. Quantitative correlations between the PVA solution concentration, coating thickness, and tribological performance have not been established, and the stress distribution mechanism of PVA coatings on aluminum substrates has not been clearly elucidated. Although stress generation and distribution in polymer coatings are directly related to coating durability, research on stress distribution in PVA-aluminum systems and their effect on tribological performance remains insufficient.^10,11^ The absence of long-term durability data under various load conditions also represents a challenge that must be resolved for practical application design guidelines.

This study aims to resolve these research gaps by optimizing pure PVA coatings on aluminum 5052 alloy substrates. The objectives of this study were to quantitatively elucidate the effects of PVA concentration and coating thickness on friction and wear characteristics, analyze the stress distribution mechanism of PVA coatings through finite element analysis, derive optimal coating conditions under various load conditions, and evaluate long-term durability. By excluding the complexity of composites and focusing on pure PVA systems, this study elucidates the fundamental tribological mechanisms of PVA coatings and presents practical design guidelines for their application.

## 2. Materials and methods

### 2.1. Materials

Polyvinyl alcohol (PVA) with a degree of polymerization of *n* = 1500 was purchased from Daejung Chemical Co. Ltd, Siheung, Korea. Aluminum 5052 alloy sheets with dimensions of 20 mm × 20 mm × 1 mm were used as substrates. Acetone, ethyl alcohol, and deionized (DI) water were obtained from Duksan Pure Chemicals Co. Ltd, Ansan, Korea.

### 2.2. Specimen fabrication

**Fig. 1:**
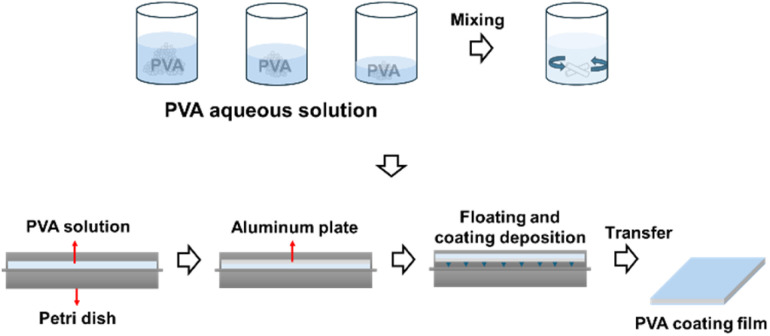
Schematic illustration of PVA coating preparation by solution casting–floating method.

PVA coatings were prepared using a solution casting–floating method (Fig. 1). PVA powder was dissolved in 100 mL of DI water at concentrations of 0.1, 1, 5, and 10 wt%. The solution was mixed at 200 rpm and heated to above 90 °C for approximately 4 h to achieve complete dissolution. The prepared PVA solution was then poured into cylindrical molds with a diameter of 40 mm and a height of 100 mm. The molds were filled to three different levels: 1/5, 2/5, and 3/5 of the total height of the mold.

Prior to coating, the aluminum substrates were cleaned sequentially in acetone, ethanol, and DI water using ultrasonic treatment for at least 10 min. The cleaned aluminum substrates were floated on the surface of the PVA solution in the molds and fixed in place. The specimens were stored in this configuration for approximately 24 h at room temperature. Subsequently, the coated specimens were dried in a convection oven at 70 °C for 3 h.

The specimen designation followed a systematic nomenclature based on the PVA concentration and mold fill level. For example, a specimen coated with a 1 wt% PVA solution at a 2/5 mold fill level was designated as “1-2L’’.

### 2.3. Experiments

Various characterization techniques were employed to evaluate the performance of PVA coatings prepared under different conditions. Fourier-transform infrared spectroscopy (FTIR; Nicolet 6700, Thermo Scientific, Seoul, Korea) was performed to analyze the chemical composition of the PVA coatings. The analysis was conducted in absorbance mode over a wavenumber range of 650–3500 cm^−1^ with a resolution of 2 cm^−1^. X-ray Diffraction (XRD; EMPyrean, PANalytical, Malvern, UK) analysis was performed on the PVA coatings. The measurements were conducted from 5° to 90° 2*θ* with a step size of 0.02°. The analysis was performed at 40 kV and 30 mA using Cu Kα radiation (1.54 Å).

Coating thickness was measured using an ultrasonic thickness gauge (UT343D, UNI-T Technology Co., Ltd., Guangdong, China) with a resolution of 0.1 μm. Measurements were taken at five different points on each specimen and averaged. Bare aluminum specimens were used as the zero-reference standard. The accuracy of ultrasonic measurements was verified by cross-sectional optical microscopy on selected samples.

The surface roughness of each coating was evaluated using a contact surface roughness tester (SV-2100M4, Mitutoyo Co., Ltd., Kawasaki, Japan). The stylus tip was brought into contact with the specimen surface under a load of 0.75 mN. A scanning distance of 5 mm was covered at a speed of 0.5 mm s^−1^, and the calculated arithmetic mean roughness (*R*_a_) values were used in the analysis.

The friction and wear characteristics of the PVA coatings with varying concentrations and thicknesses were evaluated using a reciprocating tribometer (RFW 160, NEOPLUS Co., Ltd., Daejeon, Korea). A steel ball with a diameter of 1 mm was used as the countertip. The tests were performed for 5000 cycles at a speed of 8 mm s^−1^ under an applied normal load of 50 mN. The long-term durability of the coatings prepared under optimized conditions was evaluated at loads of 50, 70, and 100 mN. These tests were performed at a speed of 8 mm s^−1^ for up to 10 000 cycles. The wear morphology formed on the surface after friction testing was analyzed using an optical microscope (DM750, Leica, Wetzlar, Germany). All experiments were performed at least five times to ensure the reliability of the results, and mean values were used in the results.

Finite element analysis was performed to analyze the stress behavior of the PVA coatings. Material properties used in the simulation were as follows: aluminum substrate had an elastic modulus of 70 GPa and Poisson ratio of 0.33, PVA coating had an elastic modulus of 1.4 GPa and Poisson ratio of 0.45, and the counter tip had an elastic modulus of 210 GPa and Poisson ratio of 0.35.^12^ Both surfaces were modeled as smooth given their low roughness (∼0.1 μm). After initial static contact under normal loads (50, 70, 100 mN), reciprocating sliding was simulated at 8 mm s^−1^ matching experimental conditions. Friction coefficients from experimental results were applied. The analysis used a step time of 0.1 s to capture stress evolution during sliding. A fine mesh size of 0.005 was used to guarantee the reliability of the simulation results. The viscoelastic behavior of PVA was simplified to a linear elastic model, which is acknowledged as a limitation of this study.

## 3. Results and discussion

**Fig. 2:**
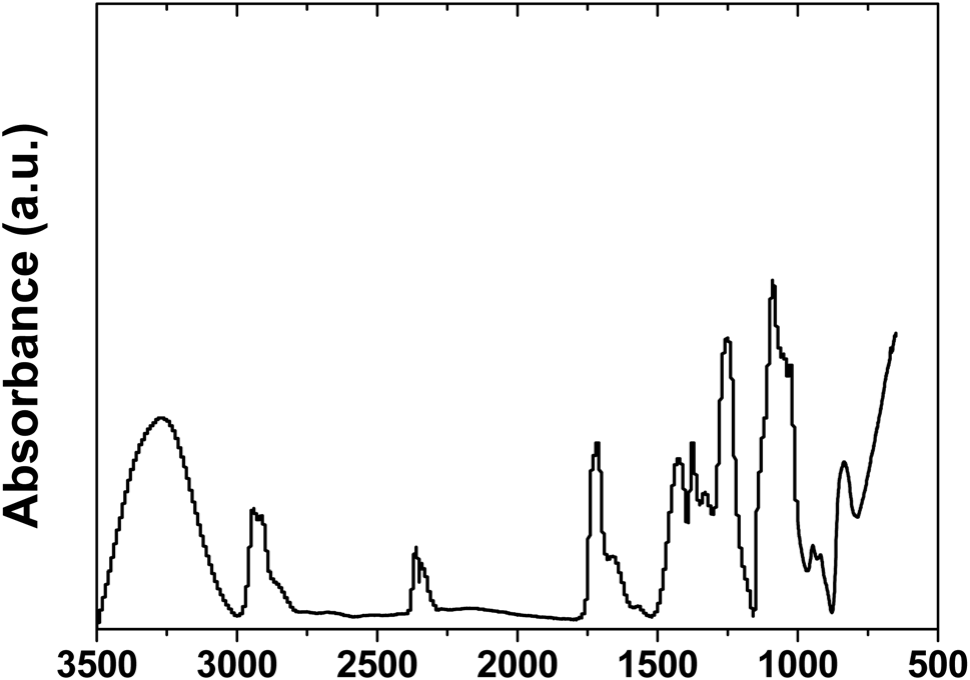
FTIR spectra of PVA film.

Fig. 2 shows the FTIR analysis results for the PVA film. Characteristic peaks were observed at 3281, 2942, 2360, 1713, 1421, 1374, 1250, 1088, 946, and 833 cm^−1^. The broad absorption band at 3281 cm^−1^ is attributed to the stretching vibration of the hydroxyl groups (–OH) in PVA.^13^ The width and position of this peak indicate the degree of hydrogen bonding between the PVA chains, which is directly correlated with the mechanical properties of the coating. The peak at 2942 cm^−1^ represents C–H stretching vibration, while peaks at 1421 cm^−1^ and 1374 cm^−1^ correspond to CH_2_ bending vibration and CH deformation vibration, respectively.^14^ The strong absorption peak at 1088 cm^−1^ is due to C–O stretching vibration, which is a major indicator for confirming the PVA backbone structure.^15^

The crystallinity index was calculated using the intensity ratio method at 1141 cm^−1^ and 1095 cm^−1^ peaks. The CI value (*I*_1141_/*I*_1095_) of 0.401 was measured. Based on the relative intensity calculation, the crystallinity was estimated to be 28.6%.^16^ The hydrogen-bonding index analysis revealed HBI = 0.594, with hydroxyl groups distributed as 59.4% intermolecular, 32.4% intramolecular, and 8.2% free OH groups.

**Fig. 3:**
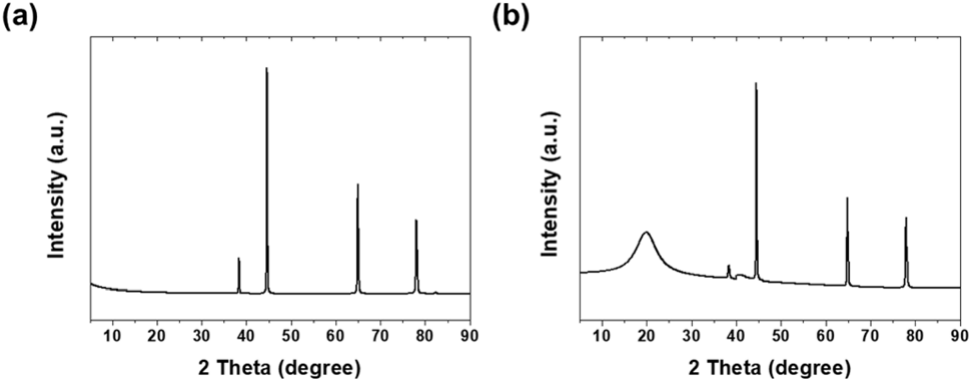
XRD patterns of (a) bare aluminum and (b) PVA-coated Al.

Fig. 3 shows the XRD patterns of bare and PVA-coated aluminum. For bare aluminum, major diffraction peaks were observed at 38.346°, 44.587°, 64.924°, and 78.021°. These correspond to the (111), (200), (220), and (311) crystal planes of aluminum, respectively, confirming the face-centered cubic (FCC) structure of aluminum.^17^ The highest intensity at 44.587° indicates the (200) preferred orientation of the Al substrate.^18^

In the XRD pattern of PVA-coated specimens revealed a broad peak at 19.88°, corresponding to the (101) reflection of PVA crystalline domains within the semi-crystalline polymer structure.^19^ This peak, with a calculated *d*-spacing of 4.46 Å, exhibited a large FWHM of 5.57°, indicating small crystallite size and imperfect crystal ordering typical of solution-cast PVA films rather than instrumental broadening.^20^ Using the Scherrer equation, the crystallite size was determined to be 1.43 nm.^21^

The crystallinity of PVA, formed through hydrogen bonding between molecular chains, plays a crucial role in enhancing the mechanical strength and wear resistance of the coating. The broad peak width and small crystallite size suggest relatively low crystallinity, consistent with the FTIR-determined crystallinity value of 28.6%. Additionally, the aluminum substrate peak intensities showed a significant reduction of 57.7–86.0% after PVA coating, demonstrating both effective polymer layer coverage and the X-ray attenuation effect of the PVA coating layer.

**Fig. 4:**
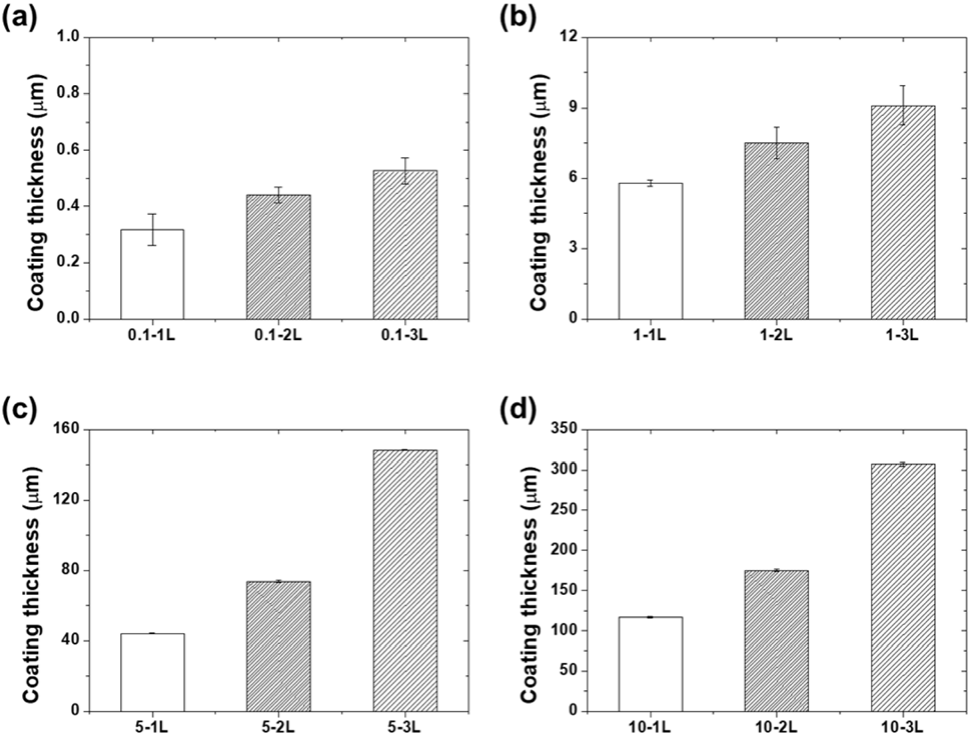
Coating thickness as a function of the mold fill level for (a) 0.1 wt%, (b) 1 wt%, (c) 5 wt%, and (d) 10 wt% PVA concentrations.

Fig. 4 shows the variation in the coating thickness according to the PVA concentration and mold fill height. Coatings prepared at a concentration of 0.1 wt% (0.1-1L, 0.1-2L, and 0.1-3L) formed very thin layers with thicknesses of 0.32, 0.44, and 0.53 μm, respectively. These ultra-thin coatings are likely to form non-uniform coverage because they cannot completely cover the surface micro-asperities of the substrate.

At 1 wt% concentration, the coating thicknesses increased to 5.8, 7.5, and 9.1 μm. This thickness range corresponds to the critical thickness at which PVA molecular chains can form sufficient networks to produce continuous films. At 5 wt% concentration, thicknesses of 44.2, 73.8, and 148.3 μm were measured, whereas at 10 wt% concentration, thicknesses reached 117, 175, and 307 μm. The increase in thickness with concentration can be attributed to the increased solution viscosity and higher solid content.^22^

**Fig. 5:**
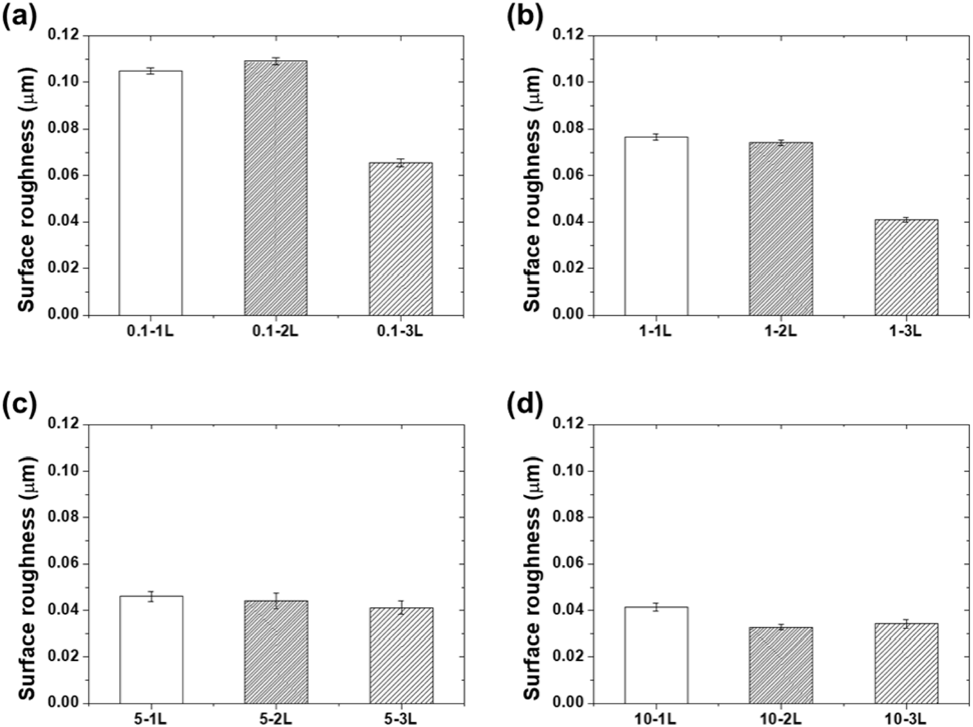
Surface roughness (*R*_a_) values for (a) 0.1 wt%, (b) 1 wt%, (c) 5 wt%, and (d) 10 wt% PVA coatings at different mold fill levels.

The surface roughness measurements in Fig. 5 demonstrate the surface-smoothing effect of the coatings. While bare aluminum had a surface roughness of 0.103 μm, most coated specimens showed a decreasing trend in surface roughness. In particular, the 10-2L and 10-3L specimens exhibited low roughness values of 0.033 and 0.034 μm, respectively. This indicates that the PVA solution filled the microasperities on the aluminum surface to form a smooth surface. The reduction in surface roughness increases the real contact area during frictional contact, which favorably affects the stress distribution.^23^

**Fig. 6:**
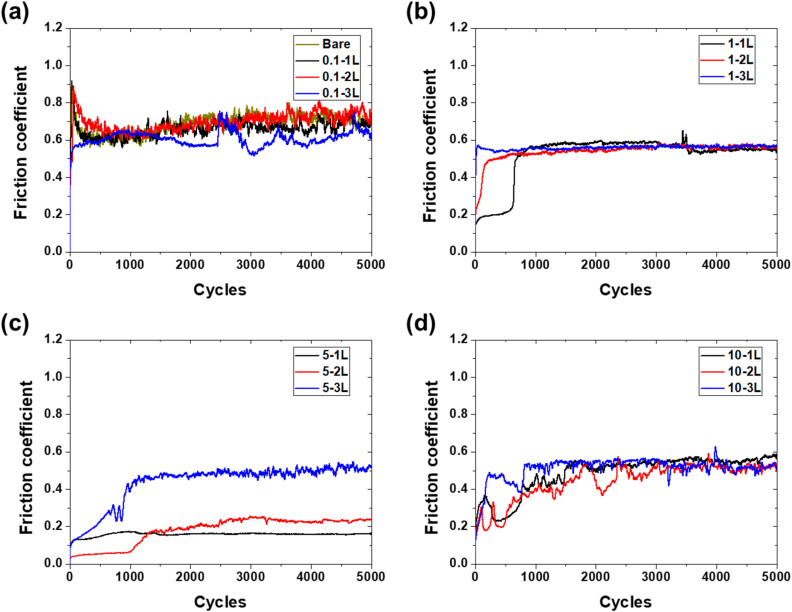
Friction coefficient evolution during 5000 cycles for (a) bare aluminum and 0.1 wt%, (b) 1 wt%, (c) 5 wt%, and (d) 10 wt% PVA coatings.

Fig. 6 shows the time-dependent variation in the friction coefficient according to the coating concentration and thickness. Bare aluminum started with a high friction coefficient of 0.9, decreased to approximately 0.6 by 1000 cycles, and maintained a range of 0.6–0.75 until 5000 cycles. The 0.1-1L specimen exhibited almost identical behavior to bare aluminum, starting at 0.9 and finally reaching a level of 0.65. The 0.1-2L specimen showed a similar pattern but displayed a slightly higher range of 0.7–0.8 after 2000 cycles. The 0.1-3L specimen started at 0.5, increased to 0.65 by 1000 cycles, sharply rose to 0.7 at 2500 cycles, and then maintained a level of 0.6 with fluctuations.

The friction coefficient of the 1-1L specimen began at a low value below 0.2 but sharply increased to 0.6 at 500 cycles, maintained a constant value until 3500 cycles, and then experienced abrupt changes and reached 0.55 at the final cycle. The 1-2L specimen increased from an initial 0.22 to 0.5 by 200 cycles and remained relatively constant until 5000 cycles. The 1-3L specimen started at 0.58 and exhibited relatively stable behavior in the range of 0.55–0.60 throughout the entire test period.

The 5-1L specimen started with a low friction coefficient of 0.13 and exhibited very stable behavior in the range of 0.13–0.16 throughout 5000 cycles. The 5-2L specimen began at a very low value of 0.05, gradually increased to 0.2 by 1500 cycles, and maintained a range of 0.20–0.23 until 5000 cycles. The 5-3L specimen increased from an initial 0.1 to 0.3 after 700 cycles, sharply increased to 0.5 after 800 cycles, and finally reached 0.52.

The 10-1L specimen started below 0.2, increased to 0.38 within 200 cycles, rose above 0.4 in the 500–1000 cycle interval, and maintained a level of 0.58 from 1500 to 5000 cycles. The 10-2L specimen showed large fluctuations in the range of 0.2–0.3 within 500 cycles, increased to approximately 0.5 by 1800 cycles, and reached 0.53 after experiencing further increases and decreases. The 10-3L specimen sharply increased to 0.5 by 200 cycles, rose to 0.55 at 750 cycles, and recorded a final value of 0.52 with large fluctuations thereafter.

**Fig. 7:**
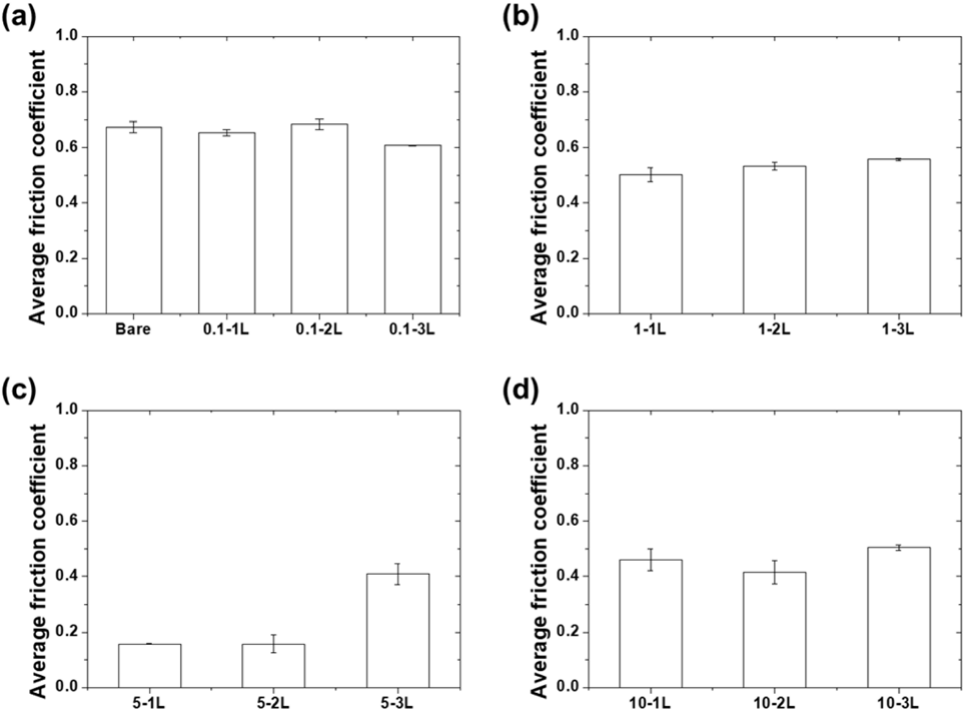
Average friction coefficients for (a) bare aluminum and 0.1 wt%, (b) 1 wt%, (c) 5 wt%, and (d) 10 wt% PVA coatings.

Fig. 7 shows the average friction coefficient values measured over 5000 cycles. The average friction coefficient of bare Al was measured to be 0.67. For coatings prepared at a concentration of 0.1 wt%, 0.1-1L, 0.1-2L, and 0.1-3L exhibited average friction coefficients of 0.65, 0.68, and 0.61, respectively, which were similar to those of bare aluminum. For the 1 wt% concentration coatings, 1-1L, 1-2L, and 1-3L recorded average friction coefficients of 0.50, 0.53, and 0.56, respectively, representing approximately 16–25% reduction compared to bare aluminum. The 5 wt% concentration coatings showed the best friction characteristics, with 5-1L and 5-2L recording very low average friction coefficients of 0.158 and 0.157, respectively, achieving approximately 76% reduction compared to that of bare aluminum. In contrast, 5-3L showed a relatively high value (0.41). For the 10 wt% concentration coatings, 10-1L, 10-2L, and 10-3L exhibited average friction coefficients of 0.46, 0.42, and 0.504, respectively, which were similar to those of the 1 wt% coatings. These results clearly demonstrate that coatings prepared at 5 wt% concentration with thicknesses ranges of 44.2–73.8 μm exhibit optimal friction characteristics.

**Fig. 8:**
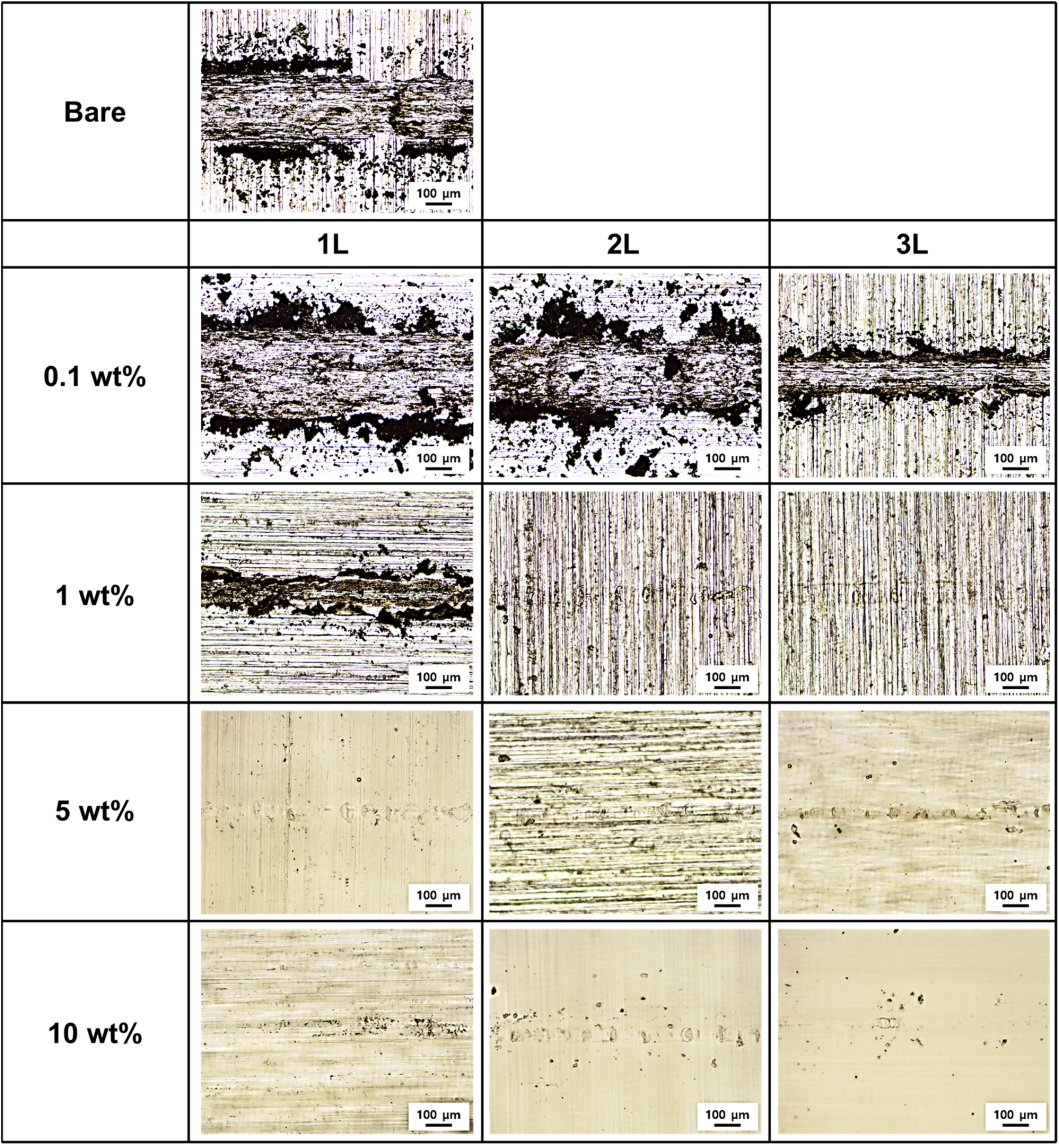
Optical microscopy images of wear tracks after 5000 cycles at a 50 mN load.

The optical microscopy images in Fig. 8 show the wear track morphologies of each specimen. Bare Al, 0.1 wt% concentration coating specimens, and 1-1L exhibited wide wear widths and severe abrasive wear patterns.^24^ Parallel scratches were observed inside the wear tracks, and several wear particles accumulated around the tracks. This represents a typical severe wear phenomenon caused by direct metal-to-metal contact.

For coatings with thicknesses greater than 1-2L, no damage to the aluminum substrate was observed. Instead, adhesive wear patterns were predominantly observed on the PVA film.^25^ During repetitive sliding contact, the PVA film was transferred to the counter ball and then re-adhered to the coating surface. This adhesive wear is related to the viscoelastic properties of PVA, and at an appropriate thickness, it serves as a sacrificial layer that effectively protects the substrate.^26^

The 5-1L and 5-2L specimens exhibited the fewest wear traces. The wear track width was narrow, and surface damage was minimized, confirming that the thickness range of 40–70 μm exhibits optimal wear resistance. Within this thickness range, PVA coatings exerted sufficient self-lubrication effects to minimize wear.

**Fig. 9:**
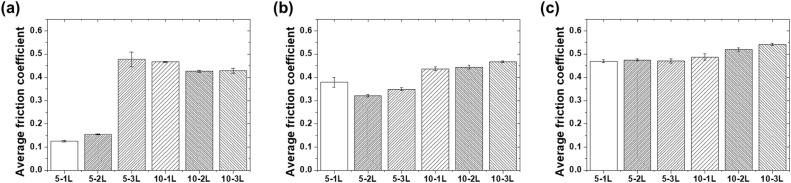
Average friction coefficients of selected coatings during 10 000 cycles under (a) 50 mN, (b) 70 mN, and (c) 100 mN loads.

Fig. 9 shows the long-term durability test results under different loads for the six selected specimens (5-1L, 5-2L, 5-3L, 10-1L, 10-2L, and 10-3L). Under the 50 mN load condition, the average friction coefficients measured during 10 000 cycles were 0.13 for 5-1L, 0.15 for 5-2L, 0.48 for 5-3L, 0.47 for 10-1L, 0.43 for 10-2L, and 0.43 for 10-3L. While 5-1L and 5-2L maintained very low friction coefficients, 5-3L and 10 wt% specimens exhibited relatively high values.

Under the increased load condition of 70 mN, 5-1L, 5-2L, 5-3L, 10-1L, 10-2L, and 10-3L exhibited average friction coefficients of 0.38, 0.32, 0.35, 0.436, 0.44, and 0.47, respectively. Although the friction coefficients increased for all specimens, the 5 wt% specimens still maintained lower values than the 10 wt% specimens. In particular, 5-2L maintained a relatively low friction coefficient of 0.32 even under a 70 mN load, demonstrating excellent self-lubricating performance.

Under 100 mN load condition, 5-1L, 5-2L, 5-3L, 10-1L, 10-2L, and 10-3L recorded average friction coefficients of 0.469, 0.475, 0.47, 0.486, 0.52, and 0.54, respectively. Under high-load conditions, all specimens converged to a similar range of 0.47–0.54, indicating that the influence of coating thickness decreased at a 100 mN load and the limitations of the PVA material became apparent. Nevertheless, 5-1L and 5-2L maintained relatively superior performances under all load conditions.

**Fig. 10:**
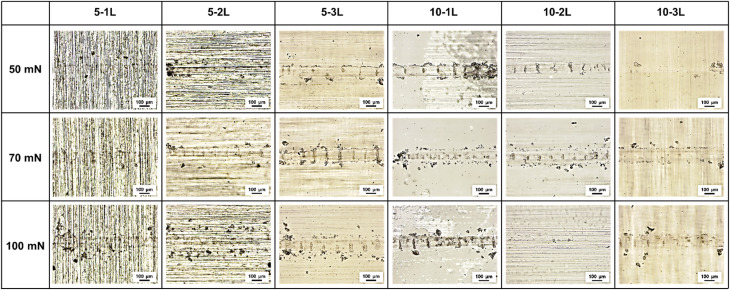
Optical microscopy images of wear tracks for selected coatings under different loads after 10 000 cycles.

The wear track analysis results by load in Fig. 10 reveal various wear mechanisms for the different loads. At a 50 mN load, 5-1L and 5-2L exhibited the narrowest wear track width and minor surface damage. Smooth surfaces caused by the plastic deformation of the PVA film were observed inside the wear tracks, indicating that the viscoelastic properties of PVA effectively absorbed the contact stress.^27^ In contrast, thick coatings beyond 5-3L formed deep grooves along the center of the wear tracks. This demonstrates that an excessive coating thickness accelerates wear by inducing an increased contact area and heat accumulation.

At a 70 mN load, the wear width increased for all specimens, but 5-1L and 5-2L still experienced wear only within the coating layer without substrate exposure. Evidence of transfer film formation was observed inside the wear tracks, indicating that PVA adhered to the counterball surface and was re-transferred to the coating surface during repetitive sliding. In the 10 wt% specimens, local delamination of the coating was observed at the center of the wear tracks, suggesting that the interfacial bonding strength of the thick coating layers weakened under repetitive loading.

Under the 100 mN load condition, more severe wear patterns were observed. Although microcracks occurred at the center of the wear tracks in 5-1L and 5-2L, the substrate was still protected. The wear mechanism appeared mainly as a combination of adhesive and fatigue wear.^28^ Adhesive wear occurred because of the strong adhesion between the PVA and counter ball, whereas fatigue wear resulted from cumulative damage due to repetitive loading. For 10-3L, severe plastic deformation across the entire wear track was observed, along with partial removal of the coating in some sections. This shows that an excessive thickness of 307 μm acts as a critical weakness under high-load conditions. Overall, 5-1L and 5-2L with thicknesses ranges of 44.2–73.8 μm exhibited the best wear resistance under all load conditions, confirming that PVA coatings perform an effective protective role within the optimal thickness range.

The long-term durability tests provide indirect evidence of strong interfacial adhesion between PVA and aluminum substrate. Despite 10 000 cycles under varying loads (50–100 mN), no coating delamination was observed in the optimized specimens (5-1L and 5-2L). The wear tracks remained confined within the coating layer without exposing the aluminum substrate, even under the highest load of 100 mN. This sustained coating integrity under prolonged cyclic loading demonstrates effective interfacial bonding achieved through the solution casting–floating method.

Table 1 compares the tribological performance of pure PVA coating with literature-reported PVA-based composite systems.^7,8,25^ Composite systems incorporating graphene oxide demonstrate lower friction coefficients (0.085 for PVA/GO and 0.05 for PVA/GO/borax). However, pure PVA coating achieves comparable durability of 10 000 cycles while eliminating the complexity associated with nanomaterial dispersion. At the same test load (50 mN), pure PVA coating shows equivalent durability to the PVA/graphene oxide composite while maintaining process simplicity. The PVA/MWCNT system exhibited higher friction (>0.3) despite carbon nanotube inclusion, indicating that nanofillers do not guarantee friction reduction. These comparisons demonstrate that pure PVA coatings can serve as effective tribological coatings without the manufacturing complexity and quality control challenges inherent in composite systems.

**Table 1:** Comparison of tribological performance between pure PVA and PVA-based composite coatings from literature

Coating system	Friction coefficient	Normal load	Test cycles/duration	Test method	Ref.
Pure PVA	0.13	50 mN	10 000 cycles	Ball-on-plate reciprocating	This work
PVA/graphene oxide	0.085	50 mN	5000 cycles	Ball-on-plate reciprocating	7
PVA/graphene oxide/borax	0.05	1 N	8 h, 576 m	Ball-on-disk reciprocating	8
PVA/multi-walled carbon nanotubes	>0.3	<8 N	10 000 cycles	Ball-on-disk reciprocating	25

**Fig. 11:**
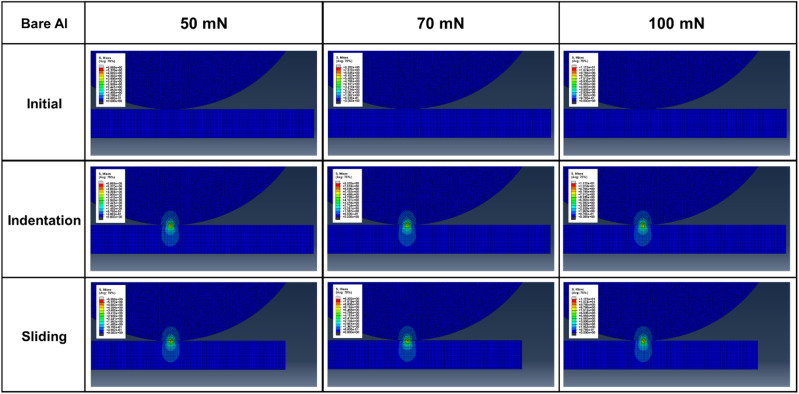
Finite element analysis results showing the von Mises stress distribution for bare aluminum.

**Fig. 12:**
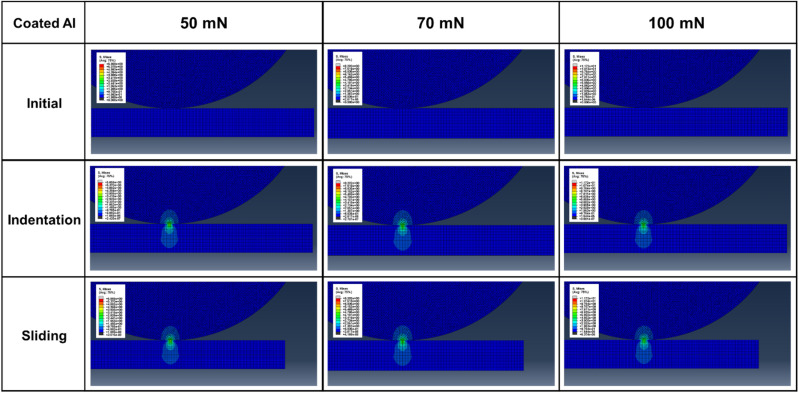
Finite element analysis results showing von Mises stress distribution for PVA-coated aluminum (5-1L).

Fig. 11 and 12 show the stress distribution results obtained through the finite element analysis. For bare aluminum, typical Hertzian contact patterns were observed, with a stress concentration at the center of the contact area. Maximum von Mises stress occurred directly beneath the contact center, suggesting the possibility of localized plastic deformation.^29^

The stress distributions of the PVA-coated specimens exhibited markedly different patterns. The stress was dispersed around the contact area and distributed over a wider region. This demonstrates that the low elastic modulus of PVA (1.4 GPa) resulted in a stress redistribution effect. PVA coatings accommodate deformation while transferring loads over a larger area, thereby reducing the maximum stress applied to the substrate.

**Fig. 13:**
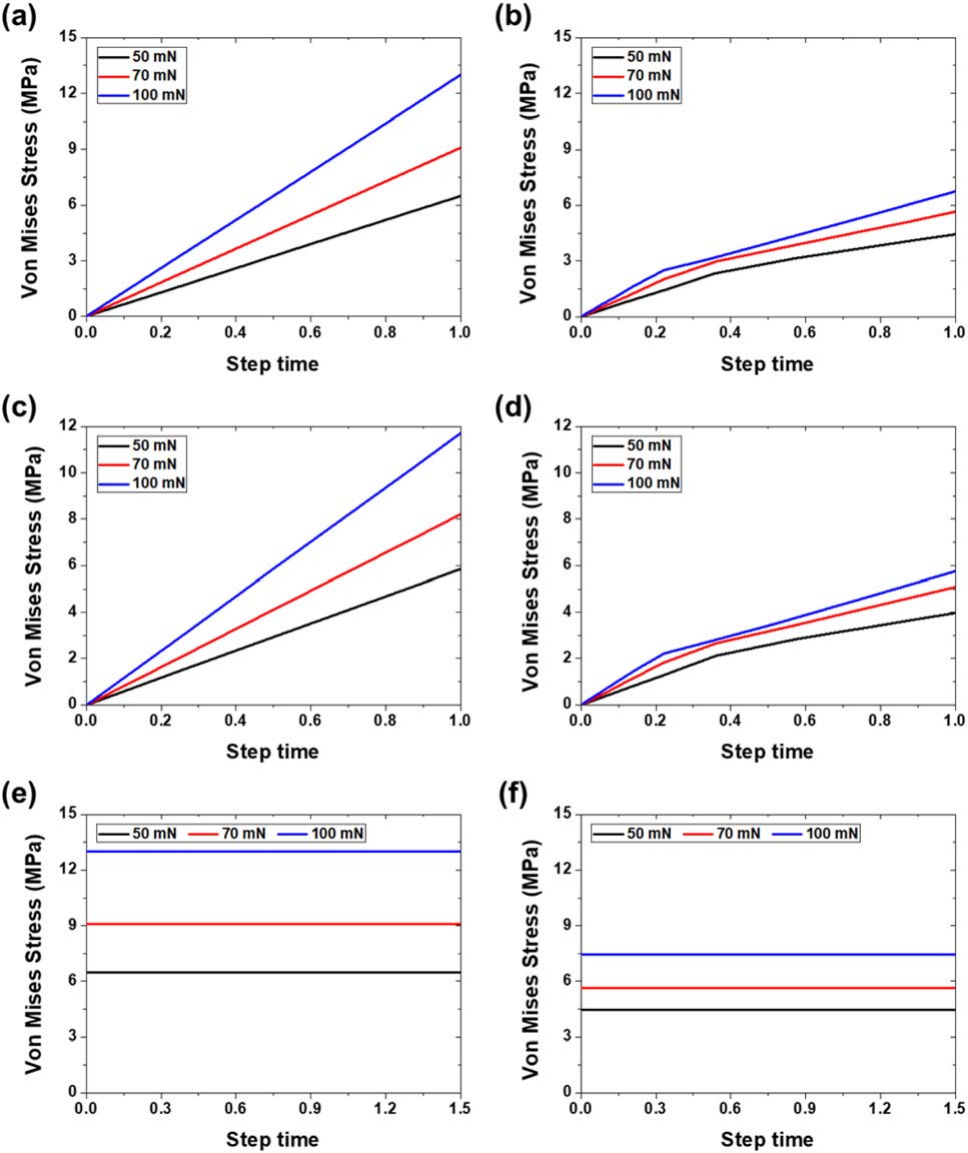
von Mises stress values from FEA: (a) counter tip stress during indentation on bare Al, (b) counter tip stress during indentation on PVA-coated Al, (c) substrate stress during indentation on bare Al, (d) substrate stress during indentation on PVA-coated Al, (e) counter tip stress during sliding on bare Al, and (f) counter tip stress during sliding on PVA-coated Al.

The quantitative analysis results in Fig. 13 show that the von Mises stress acting on the counter tip in bare aluminum was 6.49, 9.09, and 12.99 MPa under loads of 50, 70, and 100 mN, respectively. In the PVA-coated specimens, these values decreased by 31–48% to 4.45, 5.64, and 6.76 MPa under the same load conditions. The stress acting on the substrate also decreased from 5.86, 8.2, and 11.72 MPa for aluminum to 3.97, 5.07, and 5.75 MPa for the PVA coating, representing a 32–51% reduction.

The dynamic sliding simulation showed that shear stress distribution differed significantly between bare aluminum and PVA-coated specimens. During sliding, the PVA coating accommodated tangential deformation through its low elastic modulus, preventing stress concentration at the contact interface. The PVA coatings effectively absorbed shear stress, thereby minimizing stress transfer to the substrate. This stress distribution mechanism demonstrates that the PVA coatings function as mechanical protective layers beyond simple lubricating films. In particular, optimal stress distribution effects were observed under 5-1L conditions (44.2 μm thickness), which is consistent with the experimentally observed excellent friction and wear characteristics.

## 4. Conclusions

Pure PVA coatings on aluminum 5052 substrates achieved optimal tribological performance at a concentration of 5 wt% and thicknesses between 44.2 and 73.8 μm. These conditions yielded friction coefficients of 0.157–0.158, representing a 76% reduction compared to bare aluminum. The wear mechanisms transitioned from severe abrasive wear in ultra-thin coatings to effective self-lubrication in optimally thick coatings, whereas excessive thickness beyond 150 μm led to delamination failure. Finite element analysis confirmed that the elastic modulus mismatch between PVA (1.4 GPa) and aluminum (70 GPa) enabled stress redistribution, reducing Von Mises stresses by 31–51%. This mechanism was validated through long-term testing under 50–100 mN loads for 10 000 cycles, where the optimized coatings maintained superior performance despite load-dependent degradation. The success of the pure PVA coatings demonstrates that complex composite formulations are unnecessary for achieving excellent tribological performance. Simple solution casting with controlled concentration and thickness parameters enables the reproducible fabrication of protective coatings for aluminum components. These findings establish practical design guidelines, where the identified optimal range of 44–74 μm thickness at 5 wt% concentration maximizes both friction reduction and wear protection in mechanical applications.

## Author contributions

Sung-Jun Lee: conceptualization, methodology, software, validation, formal analysis, investigation, data curation, writing – original draft, writing – review & editing, visualization. Hye-Min Kwon: methodology, software, validation. Chang-Lae Kim: conceptualization, methodology, resources, writing – review & editing, supervision, project administration.

## Conflicts of interest

There are no conflicts to declare.

## Data Availability

All data supporting this study are included in the article.
